# Enhancing growth and bioactive metabolites characteristics in *Mentha pulegium* L. via silicon nanoparticles during *in vitro* drought stress

**DOI:** 10.1186/s12870-024-05313-z

**Published:** 2024-07-10

**Authors:** Hany M. El-Naggar, Amira R. Osman

**Affiliations:** 1 Department of Floriculture, Faculty of Agriculture, Alexandria University (El-Shatby), Alexandria, 21545 Egypt; 2 Department of Horticulture, Faculty of Agriculture, Damanhour University, Damanhour, 22516 Beheira Egypt

**Keywords:** Elicitation, Murashige and Skoog media, Nano silicon oxide, Secondary products, Tissue culture

## Abstract

The development and production of secondary metabolites from priceless medicinal plants are restricted by drought stress. *Mentha pulegium* L. belongs to the Lamiaceae family and is a significant plant grown in the Mediterranean region for its medicinal and aesthetic properties. This study investigated the effects of three polyethylene glycol (PEG) (0, 5, and 10%) as a drought stress inducer and four silicon nanoparticle (SiNP) (0, 25, 50, and 100 ppm) concentrations as an elicitor to overcome the adverse effect of drought stress, on the growth parameters and bioactive chemical composition of *M. pulegium* L. plants grown *in vitro*. The experiment was performed as a factorial experiment using a completely randomized design (CRD) consisting of 12 treatments with two factors (3 PEG × 4 SiNPs concentrations), 6 replicates were used for each treatment for a total of 72 experimental units.

The percentage of shoot formation was inversely proportional to the PEG concentration; for the highest PEG concentration, the lowest percentage of shoot formation (70.26%) was achieved at 10% PEG. SiNPs at 50 ppm enhanced shoot formation, the number of shoots, shoot height, fresh and dry weight, rosmarinic acid, total phenols, and 2,2-diphenyl-1-picrylhydrazyl (DPPH) scavenging activity. The methanol extract from *M. pulegium* revealed the presence of significant secondary metabolites using gas chromatography‒mass spectrometry (GC–MS). The principal constituents of the extract were limonene (2.51, 2.99%), linalool (3.84, 4.64%), geraniol (6.49, 8.77%), menthol (59.73, 65.43%), pulegone (3.76, 2.76%) and hexadecanoic acid methyl ester or methyl palmitate (3.2, 4.71%) for the 0 ppm SiNPs, PEG 0% and 50 ppm SiNPs, and PEG 10%, respectively. Most of the chemical components identified by GC‒MS in the methanol extract were greater in the 50 ppm SiNP and 10% PEG treatment groups than in the control group. SiNP improves drought tolerance by regulating biosynthesis and accumulating some osmolytes and lessens the negative effects of polyethylene glycol-induced drought stress.

Based on the results, the best treatment for most of the parameters was 50 ppm SiNPs combined with 10% PEG, the morphological and chemical characteristics were inversely proportional to the PEG concentration, as the highest PEG concentration (10%) had the lowest results. Most parameters decreased at the highest SiNP concentration (100 ppm), except for the DPPH scavenging percentage, as there was no significant difference between the 50 and 100 ppm SiNPs.

**Supplementary Information:**

The online version contains supplementary material available at 10.1186/s12870-024-05313-z.

## Introduction

More than 6000 years ago, aromatic herbs were employed in Egypt for pharmacological, cosmetic, and medical purposes [1]. The Lamiaceae family is recognized for its vital oils, medicinal uses, and antimicrobial activity. *Mentha* is an important genus of Lamiaceae [2].

**Fig. 1:**
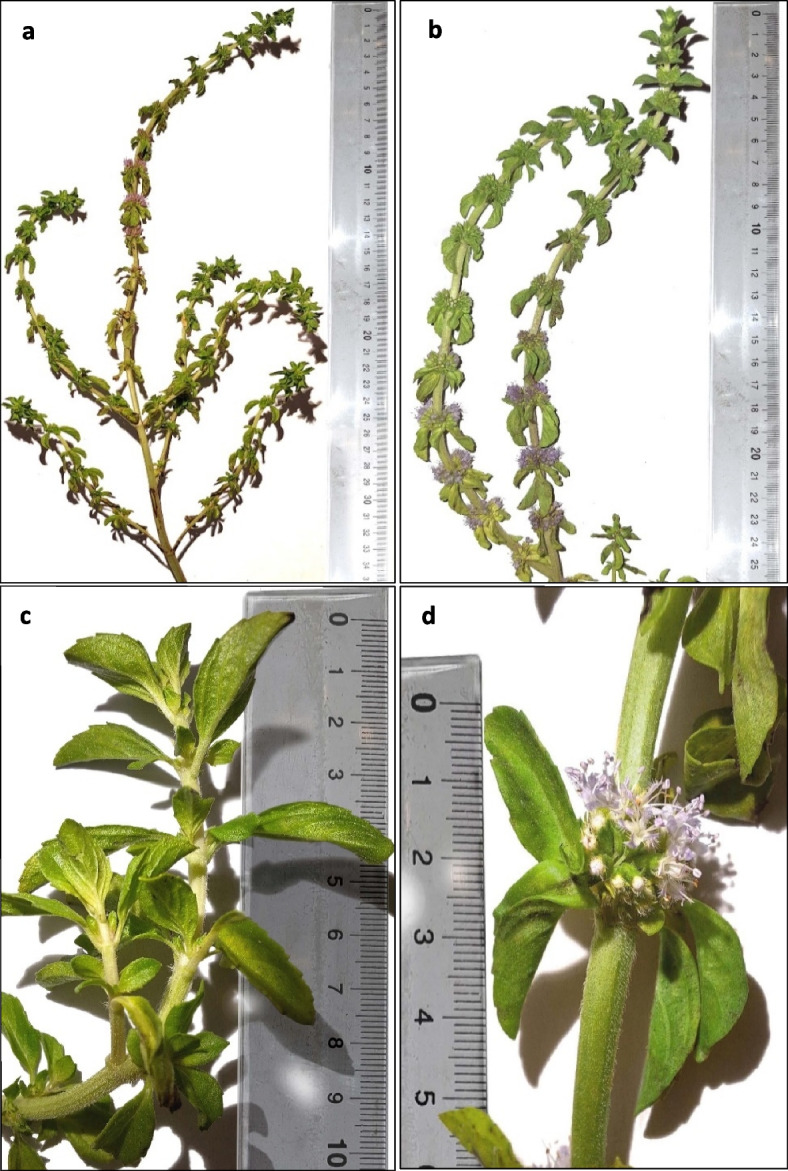
**a** *Mentha pulegium* **b** flowering branch **c** leafy branch **d** inflorescences

*Mentha pulegium* L. (commonly named pennyroyal) [1] and known in Morroco as ‘‘Feliou” [2] originated in the Caucasus, Central Asia, the Mediterranean, Asia Minor, Central Europe, and northern parts of Iran is a hairy perennial with erect and prostrate stems, heavily branched.. The leaves are oval, 8–25 mm long, 5–112 mm wide, slightly dentate, apex obtuse, long-haired, the floral leaves are sessile, shorter than the flowers. Inflorescences in axillary umbels, remote, globular, 12–15 mm broad; verticillasters numerous, many-flowered, bracts ovate. Corolla 5–7 mm long, vivid pink, rose-lilac, or white [3, 4] (Fig. 1) (Additional file 2 Fig. S1 to S3).

The *Mentha pulegium* is an infusion used in traditional medicine to prevent various stomach illnesses and respiratory tract inflammation. The yield of oil made from the aerial parts of *M. pulegium* ranged between 1 and 19% of the yield found in the various examined samples, Beghidja et al. [3] suggested that these characteristics are related to the essential oil and its components, which might change depending on the environment [5]. There have been reports of larvicidal and repellent effects of *Mentha pulegium* on several mosquito populations [6].

*Mentha pulegium*, a member of the Labiatae family, is a species of medicinal plant used in folklore that has intriguing antioxidant qualities. It is extensively utilized in the pharmaceutical, agro-alimentary, and traditional medical fields [7].

Plant phenols are important naturally occurring antioxidants that can be broadly categorized into four groups: volatile oils (eugenol, carvacrol, thymol, and menthol), phenolic diterpenes (carnosol and carnosic acid), flavonoids (quercetin and catechin), and phenolic acids (gallic, protochatechuic, caffeic, and rosmarinic acids) [8]. Identifying an herbal antioxidant for use instead of a synthetic antioxidant is a new area of study that researchers have been pursuing in the last few years due to its benefits, which include inhibiting human cancer cells [9], providing active food packaging [10], and protecting against lipid-protein oxidative deterioration [11].

There are 25–30 Mentha species found in temperate regions of South Africa, Australia, and Eurasia [12], because of its anti-inflammatory, carminative, antiemetic, diaphoretic, antispasmodic, analgesic, stimulant, emmenagogue, and anticatharrhal properties, Mentha spp. have been used as folk remedies for nausea, bronchitis, flatulence, anorexia, ulcerative colitis, and liver complaints [13, 14].

Certain secondary metabolites arise during plant growth as cells differentiate and mature in response to the activation of morphological differentiation [15]. Although plant growth and survival do not depend on these metabolic intermediate products, secondary metabolites are necessary for a plant to interact with its surroundings when under stress [16]. By regulating plant growth and development, serving as reservoirs for vital phytochemicals, and protecting plants from a range of environmental obstacles, these compounds are produced to enable plants to compete for and increase their viability in their natural habitat [17]. Consequently, techniques to improve plant resistance to drought must be identified and developed, particularly for arid and semiarid regions [18, 19].

Despite their enormous commercial value, secondary metabolites are still produced in small quantities both *in vitro* and *in vivo*. The large-scale synthesis of most commercially important bioactive metabolites from domestic or wild plants, as well as from other tissue cultures, is hampered by several issues, and elicitation has been shown to be a useful method for boosting or improving the synthesis of secondary metabolites in plants [16].

Elicitation is the process of adding biotic or abiotic elicitors externally to growth media. This causes cells, tissues, or organs to experience stress and produce additional secondary metabolites [20]. Elicitors encourage physiological and biochemical reactions that trigger the plant's defense mechanism. These compounds act as signals that are recognized by elicitor-specific receptors found on plant cell membranes; during elicitation, they cause defensive reactions that boost the synthesis and storage of bioactive metabolites [21]. Usman et al. [22] mentioned that many researchers have been interested in the ability of nanoparticles to elicit the expression of genes involved in the manufacture of secondary metabolites. NPs could be used as novel antibiotic elicitors for the production of bioactive chemicals in plant cells and tissue culture. The capacity of nanoparticles to elicit the expression of genes involved in the manufacture of secondary metabolites has drawn the attention of numerous researchers in recent years.

PEG was added to the culture media as an osmotic agent to cause drought stress in the callus culture. In plant cells, wounding and drought stress cause oxidative stress, which leads to the production of reactive oxygen species (ROS), including superoxide (O_2_), hydroxy radical (OH), hydrogen peroxide (H_2_O_2_), and alkoxy radical (RO) [23]. The disturbance of cell metabolism caused by drought stress leads to an increase in reactive oxygen species. This process is followed by damage to DNA, proteins, and lipids via oxidative stress [24]. Under mild and moderate water stress conditions, considerable reductions in height, leaf length, leaf area, and weight were observed as a result of drought stress in lemongrass plants [25].

A group of abiotic elicitors known as nanoparticles (NPs) are used in plant tissue cultures to increase the generation of secondary metabolites [26]. According to Abbasi et al. [27], metal oxide nanoparticles (MONPs), such as zinc oxide nanoparticles (ZnO NPs), silicon dioxide nanoparticles (SiO_2_ NPs), copper oxide nanoparticles (CuO NPs), and titanium dioxide nanoparticles (TiO_2_ NPs), have been proposed as possible elicitors to boost the synthesis of bioactive metabolites in *in vitro* plant cell cultures. The creation and development of nanomaterials is the focus of nanotechnology science. Because of their unique characteristics, which include their shape, chemical reactivity, competitive binding sites, and optical activity, NPs have recently attracted increased amounts of attention [28].

Numerous studies have demonstrated that silicon can lessen the impact of biotic and abiotic stressors on plants [29–31]. Due to its ability to improve the absorption of nitrogen, phosphorus, potassium, and zinc, silicon is advantageous for plant nutrition [32]. Additionally, silicon application promotes silicification at the leaf surface, limiting dryness and the transpiration rate [33].

Savvas and Ntatsi [29] revealed that the following are the main methods by which Si mediates the reduction of abiotic stressors in higher plants: silica deposition within plant tissues, which regulates nutrient and water transport inside the plants and gives leaves mechanical strength and erectness; activation of plant antioxidant systems; complexation or coprecipitation of hazardous metals with Si in soil and plant tissues; and the use of phytohormones to alter gene expression and signaling. The fibers that make up the cell wall contain silicon, which provides wall strength and resistance against infections and pests [34].

Under drought stress induced by (PEG), the *Rosa damascena* Miller explants supplemented with 100 mg/L SiO2-NPs maintained their photosynthetic parameters more than those treated with other treatments. Additionally, the proline content was higher in the 100 mg/L SiO2-NPs. SiO2-NPs also increased the ability to withstand drought conditions by decreasing H_2_O_2_ concentration and lipid peroxidation by increasing the activity of antioxidant enzymes such as catalase (CAT), peroxidase (POD), guaiacol peroxidase (GPX), and superoxide dismutase (SOD) [35]. Prior studies have highlighted the beneficial role of silicon (Si) in enhancing plant resistance to abiotic stressors, particularly those related to water and salt [36].

According to Avestan et al. [37], the development and proliferation of apple explants were enhanced by adding SiO2-NPs to the MS (Murashige and Skoog) medium. The media enriched with 50 mg/L of Si-NPs produced the greatest number of *Lavandula angustifolia* shoots and leaves per explant, also Si-NPs greatly increased the amounts of carotenoid, chlorophyll a, and b. Beta-linalool increased most (6.2 ×) in plantlets cultivated in *in-vitro* conditions after adding Si-NPs to the growth media [38].

The growth parameters, protein, and chlorophyll content of *Phoenix dactylifera* explants were reduced under 15% PEG; however, all of these parameters, along with CAT and SOD, were raised when 3.6 mM Si was added to the growing medium [39]. According to Hamayun et al. [34], the application of Si lessens the negative effects of polyethylene glycol (PEG)-induced drought stress on plant growth characteristics, such as stem length, root weight, and chlorophyll content. It appears that applying silicon may therefore be able to lessen the negative effects of drought stress on crop plants. However, the intake and effectiveness of regular elements can be enhanced by using nanocompounds.

In the current work, we investigated the effects of adding SiNP as an elicitor to *M. pulegium* tissue culture medium to lessen the adverse impact of PEG-induced drought stress. We also examined the *in vitro* plantlets of *M. pulegium* growth and multiplication parameters, photosynthetic pigment concentration, bioactive chemical composition, and antioxidant activity to determine the best combination of PEG and SiNPs for *M. pulegium in vitro* production. Furthermore, a GC–MS analysis of the methanolic extracts obtained from plantlets cultivated in conditions containing or lacking PEG and nanoparticles has been conducted.

## Materials and methods

### Plant materials

The current study was carried out twice, from March to December 2023, at the Tissue Culture and Biotechnology Laboratory, Department of Floriculture, Faculty of Agriculture, Alexandria University, Egypt. *Mentha pulegium* L. was identified and verified according to Tackholm [40] and El-Ghorab [41], the authors identified the species and had Professor Dr. Mohamed Gamal Eltorky, Professor of Ornamental Plant Breeding at Alexandria University, Egypt, verify it, the plants were collected at the beginning of the flowering stage from different areas of the El-Beheira Governorate, Egypt (30″53′30, 87″ N and 30″41′29, 77″ E). The plants were kept in the Nursery of the Department of Floriculture, Faculty of Agriculture, Alexandria University, Egypt, from which explants were subsequently obtained.

#### Origin and sterilization of explants

The *Mentha pulegium* nodes, the fourth or fifth nodes from the growing point, were removed and used as explants Additional file 2 Fig. S4. To eliminate any foreign objects (dirt or soil), the nodes were first completely cleaned for ten minutes under running tap water. After the explants were washed for ten minutes with a household detergent, they were treated with the fungicide 5% Benlate (Benomyl 50% WP 17,804–35–2; Awiner Biotech Co. Ltd., Yuhua District, Shijiazhuang City, China) for ten minutes, followed by surface sterilization treatment with 15% sodium hypochlorite bleach (Chemajet Chemical Co. Alexandria, Egypt) for fifteen minutes and finally rinsing three times with sterilized distilled water to remove any remaining sodium hypochlorite bleach.

#### Micropropagation stage and culture conditions

*M. pulegium* nodes were inoculated on 4.43 g/L MS with vitamins supplemented with 30 g/L sucrose and 7 g/L agar solidified media (MSP09-50 L; Caisson Labs, Smithfield, UT) [42]. Two plant growth regulators, 1.0 mg/L 6-benzylaminopurine BAP and 0.5 mg/L naphthaleneacetic acid NAA; three PEG-6000 (L26080) (El-Gomhouria Co. For Trading Drugs, Chemicals and Medical Supplies, Alexandria, Egypt) concentrations (0%, 5%, and 10% w/v) and four SiNP concentrations (0.0, 25.0, 50.0, and 100.0 ppm), were tested. The pH was adjusted to 5.8 (211 Hanna Instruments, Cluj-Napoca, Romania), and media were poured into tubes measuring 2.5 cm in diameter and 15 cm in height. Media were subsequently autoclaved for 20 minutes at 121 ± 1°C with a pressure of 1.5 bar cm^–2^ (Daihan Labtech Co., Ltd., model LAC-5082SE, Namyangju City, Kyonggi-Do, Korea) [43]. The plants were grown in tubes, maintained under controlled environmental conditions in the culture room, and exposed to cool white fluorescent light with an intensity ranging from 66 to 52 μmol m^2^ sec^−1^, relative humidity of 80%, and light/dark photoperiods for 16 and 8 h, respectively.

#### Determination of morphological characteristics

The percentage of shoot formation, days of shoot formation, number of shoots, and shoot height were detected. The fresh and dry weights (FW and DW respectively) of the plants were measured with a digital scale with an accuracy of 0.001 g (Setra BL-410 Precision Balance, USA).

#### Preparation of the methanol extract

With a few modifications, the extraction process followed the methodology of Bozorgi et al. [44] and Yacob et al. [45], the leaves were dried for approximately 72 hours at 40°C in an oven. After the dried plant material was ground into a fine powder, 100 ml of 95% methanol was used to suspend 20.0 gm of the powder. The methanol was evaporated to concentrate the resultant solution, which was then incubated for one hour at 50 to 60°C. Centrifugation was used to purify the extract for five minutes at 3000 rpm instead of using the Whatman No.1 filter paper.

#### Photosynthetic pigment concentrations in fresh leaves

Samples of fresh leaves (0.1 g) were placed in 5 ml of N,N-dimethyl formamide solution and incubated overnight at a cool temperature (4–5°C). Chlorophyll a and b were measured using a spectrophotometer (Unico W49376 Spectrophotometer 1200, China) at 647 and 663 nm, respectively. Chlorophyll contents were calculated (mg/g fresh weight) according to the equations described by [46].

#### Rosmarinic acid detection

The extraction technique for rosmarinic acid (RA) followed the guidelines provided by Komali and Kalidas [47] and Lopez-Arnaldos et al. [48]. For the RA extraction, 200 mg of leaf tissue was homogenized in 10 ml of 50% methanol using a Bio-Homogenizer M 133/128–0. The mixture was heated to 55°C for two hours and centrifuged for ten minutes at 3,500 rpm. Using a Unico W49376 Spectrophotometer 1200 (Shanghai, China), the absorbance at 333 nm was measured after one ml of the extract was diluted with nine ml of 50% methanol, after which the content of rosmarinic acid was determined.

#### Total phenolic concentration

**Fig. 2:**
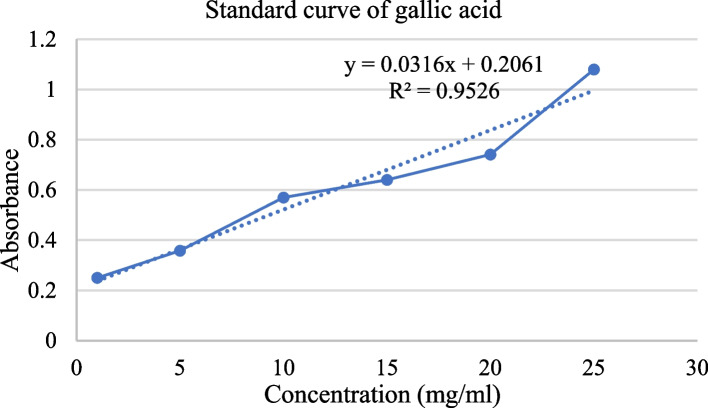
Standard curve of gallic acid (TPC)

Using the Folin‒Ciocalteu method, the total phenolic content (TPC) of the methanolic extract of *M. pulegium* leaves was determined. The methanol extract (0.2 ml, 100.0 μg·ml^−1^) was combined with 2.0 ml of Folin‒Ciocalteu reagent (diluted 1:10 with distilled water). A saturated NaHCO_3_ solution (1.5 ml, 60 g/L distilled water) was added after 5 min. All the solutions were allowed to stand for 90 min at room temperature. A spectrophotometer (Unico W49376 Spectrophotometer 1200, Shanghai, China) was then used to measure the absorbance at 725 nm. The total phenolic content was expressed as milligrams of gallic acid equivalents (GAE) per gram of dried extract [49], to create the standard gallic acid curve, standard solutions of gallic acid were prepared by diluting in methanol at 1, 5, 10, 15, 20, and 25 mg/ml (Fig. 2) Additional file 1 (Table S1).

#### Antioxidant assay and DPPH scavenging activity

The ability of the extract to scavenge radicals against stable DPPH was examined using spectrophotometry. A spectrophotometer was used with a blank composed of 3 ml of 95% aqueous methanol without DPPH and the methanol extract to provide null corrections. DPPH releases hydrogen when it interacts with an antioxidant, reducing it. The change in color from deep violet to dazzling yellow happened at 517 nm. A total of 1.5 ml of methanolic leaf extract and 1.5 ml of 0.1 ml of DPPH solution made with 95% methanol were combined. The mixture was well mixed and kept at 4°C in the dark before use. The absorbance of the resultant solution was subsequently measured at 517 nm [50, 51].

Scavenging activity (%) = (1 − absorbance of sample at 517 nm/absorbance of control at 517 nm) × 100.

Antiradical activity (DPPH) (%) = [(absorbance of control − absorbance of sample)/absorbance of control] × 100.

#### GC‒MS analysis

Using an AI-3000 autoinjector in split injection mode (1:30), a diluted volume of 1.00 μL was injected into the system; the injector temperature was 250°C. An ultratrace G.C. ISQMS from Thermo Fisher (Dreieich, Germany) was used for the test, and it had a 30-m-long TG5sil/ms capillary column. The carrier gas utilized in the experiment was helium, with a total flow of 1.5 ml (1.5 ml·min^−1^). Programmed temperature changes for the oven included 40°C for one minute, 180°C for one minute, and 200°C for two minutes. The ISQ mass spectroscopic test took 50 minutes to complete in total; the ion-source temperature was 230°C through the transfer line (250°C). The detector gain was 0.70 kV, and the solvent cutoff time was 2.00 min. Using the NIST Mass Spectral Library and the Retention Index Database, compounds were identified by their mass spectra and retention indices. The data were processed using the GC–MS Insight Xcalibur software suite [52].

#### Procedure for nano-*SiO* synthesis

**Fig. 3:**
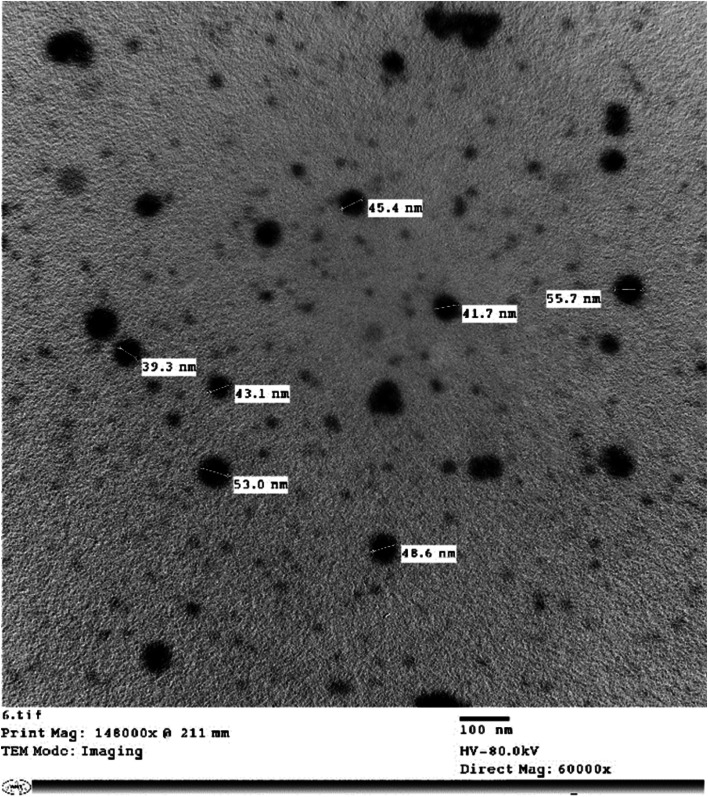
Scanning electron microscopy (SEM) image of SiO nanoparticles at a magnification of 60000x

Based on a previously published procedure by Nour et al. [53], rice husk silica nanoparticles (RH-SNPs) were generated. Rice husk was added to an alumina crucible and heated for three hours at 700°C at a rate of 20°C per minute in a muffle furnace. Following that, deionized (DI) water was subsequently used to wash the recovered silica, which was subsequently dried at 150°C. Subsequently, the silica particles underwent an hour-long mortar grinding process before being sieved to produce RH-SNPs (Fig. 3).

#### Experimental design and statistical analysis

This study aimed to induce *in vitro* drought stress in *M. pulegium* L. plants using PEG 6000 to enhance its secondary metabolites and regulate it with SiNP. The *in vitro* plantlets of *M. pulegium* growth and multiplication parameters, photosynthetic pigment concentration, bioactive chemical composition, and antioxidant activity were examined to determine the best combination of PEG and SiNPs for *M. pulegium in vitro* production. For this purpose, a factorial experiment with two factors was conducted using a completely randomized design. The first factor (main effect) was the three PEG concentrations (0%, 5%, and 10%) while the second (sub-effect) was the four concentrations of SiNP (0.0, 25.0, 50.0, and 100.0 ppm) with six replicates for each treatment, a total of 72 experimental units was used.

All the collected data were subjected to analysis of variance (ANOVA) using SAS software (version 9.5.38) to compare the different treatments. At the LSD 0.05 level, mean values for several comparison ranges were compared using Tukey's test.

## Results

### Morphological characteristics

Table 1 shows the main effects of PEG and SiNPs on the percentage of shoot formation, days of shoot formation, number of shoots, shoot height, and fresh and dry weight. The percentage of shoot formation was inversely proportional to the PEG concentration, as the highest PEG concentration (10%) had the lowest percentage of shoot formation (70.26%), while the application of SiNPs enhanced shoot formation, reaching its peak (88.5%) at 50 ppm SiNPs, after which the percentage of shoot formation was reduced at the highest silicon concentration (100 ppm).

**Table 1:** The main effects of polyethylene glycol (PEG) and silicon dioxide nanoparticles (SiNPs) on the morphological and chemical composition of *Mentha pulegium* L.treatments

	% of shoot formation	Days for shoot formation	Number of shoots	Shoot height (cm)	FW (mg)	DW (mg)	Chlo a (mg/g FW)	Chlo b (mg/g FW)	RA (mg/gm FW)	TPC (mg/g)	DPPH %
**The main effect of PEG**											
**0%**	80.81 A 77.09 A 70.26 B	21.75 C 23.42 B 25.00 A	2.83 A 2.58 A 1.75 B	7.92 A 7.63 B 6.94 C	1114.68 A 1099.44 A 876.82 B	117.50 A 116.67 A 88.68 B	0.83 B 0.93 A 0.67 C	0.33 A 0.36 A 0.18 B	0.013 B 0.027 A 0.025 A	13.09 B 15.22 A 13.88 B	38.79 B 52.25 A 52.88 A
**5%**											
**10%**											
**LSD**_**0.05**_	3.69	0.88	0.31	0.24	38.56	6.11	0.037	0.027	0.0038	1.28	3.37
**The main effect of SiNP**											
**0 ppm**	69.98 BC 77.71 B 88.50 A 68.02 C	27.00 A 21.88 C 19.44 D 25.22 B	1.89 C 2.22 BC 3.00 A 2.44 B	6.92 C 7.19 BC 8.46 A 7.41 B	992.50 B 1029.23 B 1243.72 A 855.80 C	106.84 B 109.02 B 131.14 A 83.44 C	0.75 BC 0.79 B 0.96 A 0.72 C	0.25 C 0.29 B 0.38 A 0.23 C	0.022 B 0.023 B 0.029 A 0.013 C	12.20 C 15.19 B 18.79 A 10.07 D	41.93 B 45.14 B 53.20 A 51.63 A
**25 ppm**											
**50 ppm**											
**100 ppm**											
**LSD**_**0.05**_	4.69	1.11	0.39	0.31	48.96	7.75	0.046	0.034	0.0048	1.62	4.28

*LSD*_0.05_ = least significant differences at 0.05 probability. Means with the same letters in the same column are not significantly different (*P* ≤ 0.05) according to Tukey’s test

The number of days for shoot formation was proportional to the PEG concentration, as the number of days increased with increasing PEG concentration, reaching 25 days at 10%, compared with the control (21.75 days only). On the other hand, the number of days was significantly lower in the 50 ppm SiNP treatment group (19.44 days), and there was a significant difference between the control and 100 ppm silicon treatment groups (27.0 and 25.22 days, respectively).

The number of shoots, shoot height, and fresh and dry weights were reduced at the highest PEG concentration (10%), but there was no significant difference between the control and 5% PEG treatment groups except for shoot height. While 50 ppm SiNPs significantly enhanced the number of shoots, shoot height, FW, and DW, all the other parameters decreased at the highest SiNP concentration (100 ppm).

**Fig. 4:**
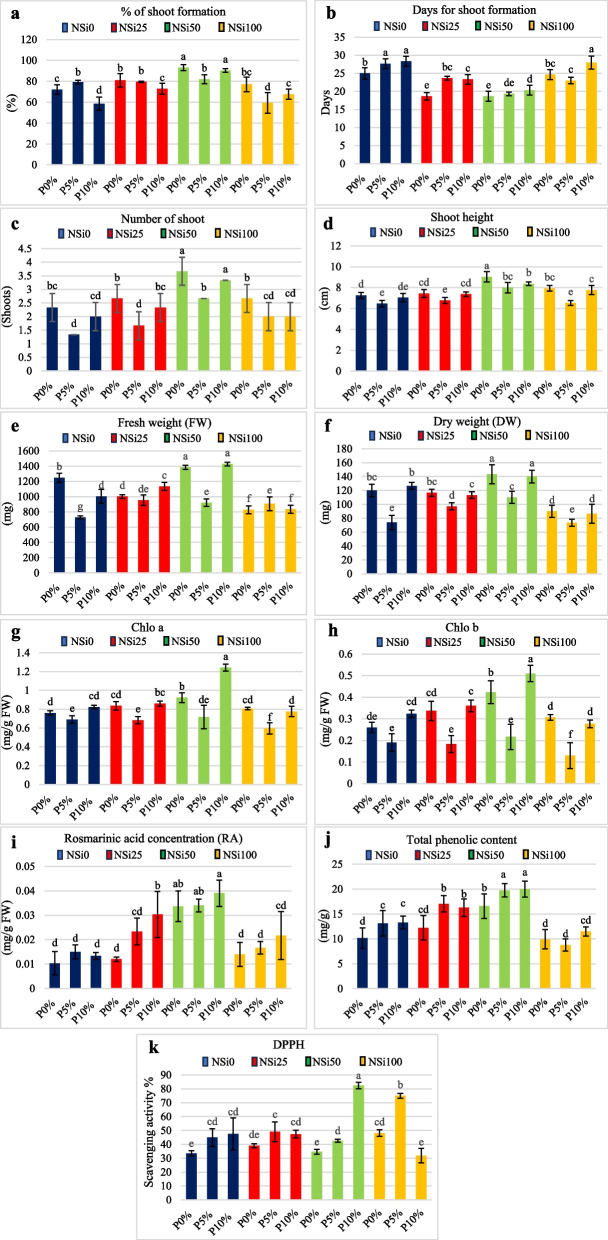
The interaction effects of PEG and SiNPs at different concentrations on the morphological and chemical characteristics of *M. pulegium*. LSD 0.05 = least significant difference at 0.05 probability. Means with the same letters are not significantly different at *p* ≤ 0.05 according to Tukey’s test. whereP0%, P5%, P10%, and NSi0, NSi25, NSi50, NSi100are the three PEG concentrations (0%, 5%, and 10%), and the four SiNP concentrations (0.0, 25.0, 50.0, and 100.0 ppm), respectively

Figure 4a to f and Additional file 1 (Table S2) show the interaction effects of PEG and SiNPs on the morphological characteristics of *M. pulegium*. The treatments with 50 ppm SiNPs combined with 0 or 10% PEG had the highest percentage of shoot formation, reaching 93.16% and 90.33%, respectively. The lowest percentage of shoot formation was achieved with the highest percentage of PEG (10%) combined with SiNPs at 0 and 100 ppm.

**Fig. 5:**
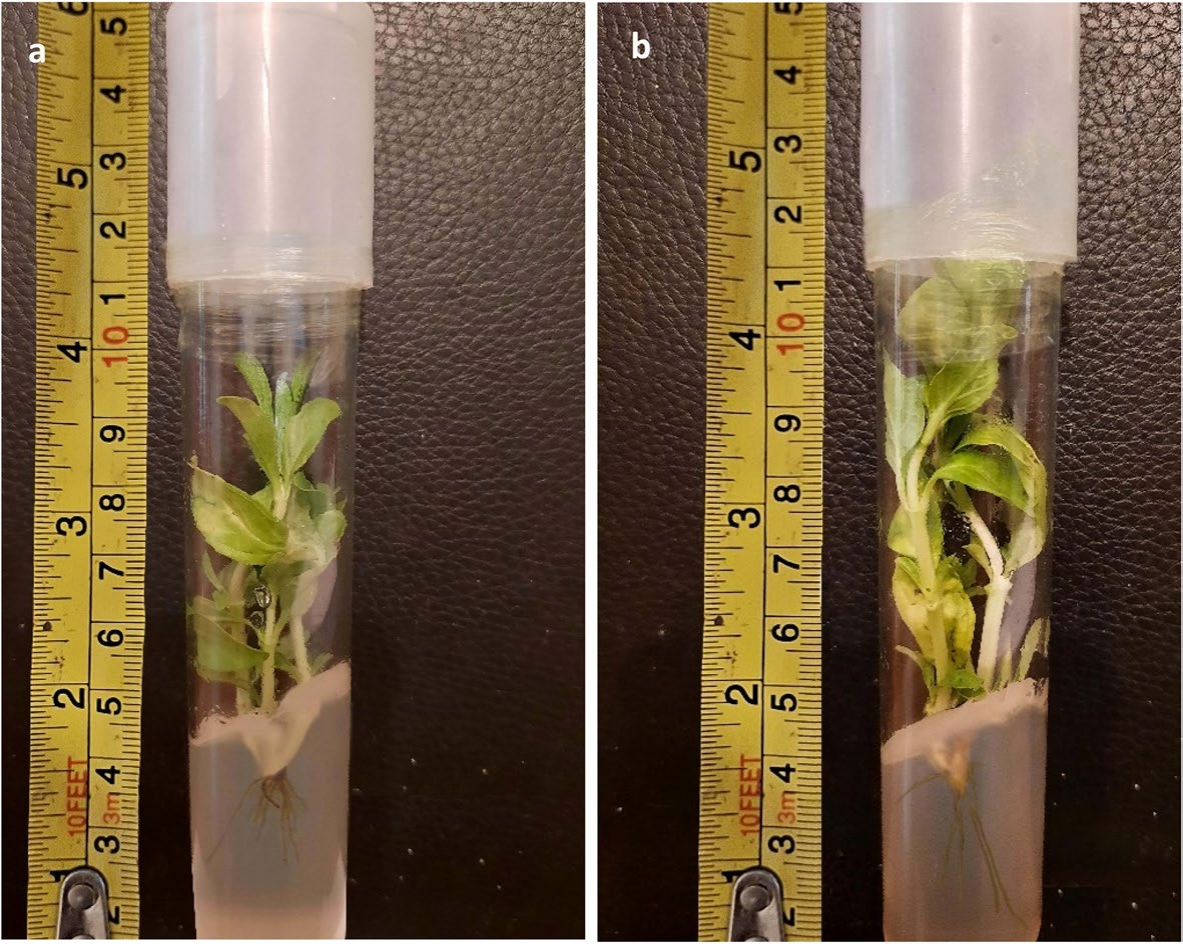
*Mentha pulegium* (**a**) 0% PEG, 0 ppm SiNPs (control), and (**b**) 10% PEG, 50 ppm SiNPs

The least number of days for shoot formation was achieved with 0% PEG combined with SiNPs at 25 and 50 ppm (18.6 days), while 10% PEG and SiNPs at 0 ppm increased the number of days needed for shoot formation to reach 28.3 days. The highest number of shoots was reached at 50 ppm SiNPs combined with 0.0 and 10% PEG (3.66 and 3.33 shoots/plant, respectively). The shoot height was also greater in the 50 ppm SiNP treatment group than in the control group (Fig. 5a and b) (Additional file 2 Fig. S5).

Both FW and DW were enhanced by the addition of SiNPs at 50 ppm combined with 0 and 10% PEG (1383.83 and 1427.16 mg FW), respectively, while both fresh and dry weight decreased significantly at the highest SiNP concentration of 100 ppm at all PEG concentrations.

#### Chemical characteristics

Figure 4g to k and Additional file 1 (Table S2) show the interaction effects of PEG and SiNPs on the chemical characteristics of *M. pulegium*. The chlorophyll a and b contents, RA content, total phenols, and DPPH scavenging activity increased from the control to 5% PEG and then decreased at 10% PEG (Table 1); however, there were no significant differences between the 5% and 10% PEG groups in terms of the RA content or DPPH radical scavenging activity. The highest SiNP concentration significantly reduced the RA and TPC, while there was no significant difference in DPPH scavenging activity between 50 and 100 ppm SiNPs; the highest RA, TPC, and DPPH were detected at 50 ppm SiNPs. For the interaction between PEG and SiNPs, the RA content and TPC increased at 50 ppm SiNPs combined with 10% PEG, while the highest concentration of SiNPs (100 ppm) significantly reduced the RA and TPC content at all PEG concentrations. The scavenging activity increased with increasing SiNP concentration at 50 and 100 ppm (Table 1) and reached its peak when SiNPs were combined with PEG at a concentration of 10% (82.37) (Fig. 4k).

**Fig. 6:**
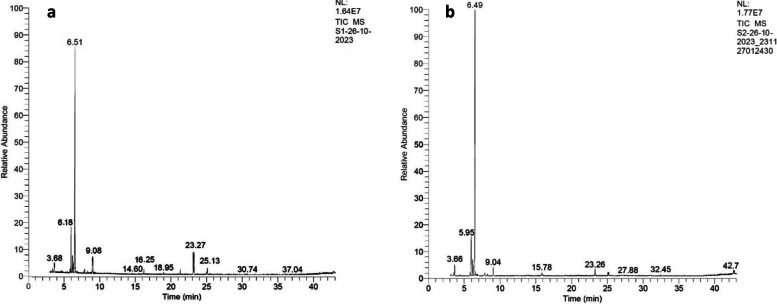
GC–MS chromatograms of *M. pulegium* (**a**) 0% PEG and 0 ppm SiNPs(control) and (**b**) 10% PEG and 50 ppm SiNPs

Using GC–MS, the chemical composition of the methanolic extract of *in vitro*-produced *M. pulegium* leaves was assessed. Table 2 and Fig. 6 display the mass spectra and relative retention durations of the extracts that were included in the data library. These data showed that 22 compounds were detected by GC/MS and accounted for approximately 97.1% of the total methanol extract. The principal constituents of the extract were limonene (2.51–2.99%), linalool (3.84–4.64%), geraniol (6.49–8.77%), menthol (59.73, 65.43%), pulegone (3.76, 2.76%) and hexadecanoic acid methyl ester or methyl palmitate (3.2, 4.71%) for 0 ppm SiNPs, 0% PEG and 50 ppm SiNPs, and 10% PEG, respectively.

**Table 2:** The phytochemical constituents of the methanol extract of *Mentha pulegium* after treatment with 0% PEG, 0 ppm SiNPs, (control), and 10% PEG, 50 ppm SiNPs

S.l no	RT 0% PEG, 0 ppm SiNP	KI 0% PEG, 0 ppm SiNP	RT 10% PEG, 50 ppm SiNP	KI 10% PEG, 50 ppm SiNP	Compound name	Molecular formula	Molecular weight	Peak area % 0% PEG, 0 ppm SiNP	Peak area %, 10% PEG, 50 ppm SiNP
1	–	–	3.12	723	Hexanoic acid	C_6_H_12_O_2_	116	–	0.47
2	3.61	661	3.59	727	Limonene (monoterpene)	C_10_H_16_	136	2.51	2.99
3	3.68	745	3.66	650	Linalool (monoterpene)	C_10_H_8_O	154	3.84	4.64
4	–	–	5.81	769	Santene	C_9_H_14_	122	–	0.7
5	6.18	752	5.95	719	Geraniol (monoterpenic alcohol)	C_10_H_8_O	154	6.49	8.77
6	6.51	653	6.49	554	Menthol (monoterpenic alcohol)	C_10_H_20_O	156	59.73	65.43
7	–	–	6.7	769	Pinane (monoterpene)	C_10_H_18_	138	–	0.36
8	–	–	6.7	738	Citronellyl propionate	C_13_H_24_O_2_	212	–	0.36
9	7.89	670	7.86	763	Camphor (monoterpene)	C_10_H_16_O	152	0.36	0.76
10	9.08	593	9.04	670	Pulegone (monoterpene)	C_10_H_16_O	152	3.76	2.76
11	16.25	671	–	–	Xanthine	C_5_H_4_N_4_O_2_	152	0.57	–
12	19.19	409	15.78	327	1-Heptacosene	C_27_H_54_	378	0.25	1.06
13	23.27	690	23.26	684	Hexadecanoic acid methyl ester or Methyl palmetate	C_17_H_34_O_2_	270	3.20	4.71
14	25.13	808	23.34	771	Heptanoic acid	C_7_H_14_O_2_	130	0.60	0.63
15	–	–	27.0	523	10-Undecanol	C_11_H_20_O	168	–	0.75
16	32.45	465	39.98	519	Cetoleic Acid	C_22_H_42_O_2_	338	0.55	0.51
17	–	–	40.64	530	Hexacosane	C_26_H_54_	366	–	0.27
18	40.86	417	41.49	426	1-Monolinoleoylglycerol trimethylsilyl ether	C_27_H_54_O_4_Si_2_	498	0.29	0.36
19	41.52	632	41.72	677	Farnesol or 2,6,10- Dodecatrien-1-o1-3,7,11-trimethyl	C_15_H_26_O	222	0.26	0.31
20	41.7	564	41.78	586	Oleic acid	C_18_H_34_O_2_	282	0.34	0.33
21	–	–	41.83	462	Ajmaline	C_20_H_26_N_2_O_2_	326	–	0.26
22	–	–	42.54	551	9-Tetradecenal, (Z)-	C_14_H_26_O	210	–	0.60

*RT* retention time

*KI* Kovats index

## Discussion

In this study, we highlighted the significant positive impacts of all documented growth and multiplication parameters, photosynthetic pigment concentration, bioactive chemical composition, and antioxidant activity when *M. pulegium* under *in vitro* production was treated with SiNP as an elicitor to mitigate the negative impact of PEG-induced drought stress and to evaluate the best combination of PEG and SiNPs for *M. pulegium in vitro* production.

Hosseini et al. [54] reported that, in prior research, the generation of secondary metabolites at a certain concentration has been shown to benefit from the use of PEG as an elicitor, with detrimental effects on the physiological and morphological properties of *in vitro* callus cultures or plantlets.

When the PEG concentration was increased to 10% in comparison to that in PEG-free media, the percentage of *in vitro* germinated *Salvia leriifolia* decreased. The PEG-free medium showed a maximum germination percentage of 89%, while the medium containing 10% PEG showed a minimum germination percentage of 65% [54]. Similarly, in our study, the percentage of shoot formation was inversely proportional to the PEG concentration, as the highest PEG concentration (10%) was associated with the lowest percentage of shoot formation (70.26%).

The detrimental effects of employing PEG *in vitro* were due to the reduction in water potential in the cultivation media of *S. lerrifollia*, chickpea varieties, and barley, and as a result, germination was greatly inhibited by increasing the PEG concentration to 10% in *S. lerrifollia* and barley and to 20% in chickpea varieties [54–56].

According to Hellal et al. [56], the disruption of cell division and elongation processes caused by a decrease in the cell's ability to absorb water and the turgescence pressure for cell enlargement may be the reasons for the decreases in most morphological properties, including stem length, stem diameter, leaf area, and total chlorophyll, when the PEG concentration is increased.

Previous results by Sarmadi et al. [57] revealed a strong correlation between the PEG concentration in culture media and the levels of malondialdehyde (MDA), a measure of lipid peroxidation, and H_2_O_2_, which serve as markers of oxidative stress in plantlets. Thus, as the PEG content in the culture media increased, an accumulation of reactive oxygen species (ROS) was observed in the plantlets of *Taxus baccata*. The disruption of the electron transport chains in the organelles involved in photosynthesis and respiration, as well as the cells' plasma membrane, may have led to this accumulation; one common effect of plasma membrane breakdown is an increase in the MDA concentration. Similar findings were obtained when growth factor levels, particularly FW, were reduced in conjunction with a sharp drop in osmotic pressure, which caused a water deficit in the plant tissues [54]. Similarly, studies have documented the detrimental effects of increased PEG concentrations on the morphological, physiological, and phytochemical characteristics of plantlets of *Tagetes erecta* [58], *Hordeum vulgare* [56], and *Allium hirtifolium* [59] cultured *in vitro*. According to our results, most of the morphological properties of *M. pulegium* decreased significantly with increasing PEG concentration which is consistent with the previous findings.

The secondary metabolite terpenoids were found in greater quantities within the culture treated with PEG [23]. Similarly, the RA content and total phenols increased with increasing PEG concentration. The RA concentration increased with increasing PEG and SiNP concentrations, reaching its peak at a SiNP concentration of 50 ppm combined with PEG at 10%, and the antioxidant activity measured by the DPPH method showed that the highest proportion of antioxidant scavenging activity was achieved at a SiNP concentration of 50 ppm combined with PEG at 10%. The DPPH assay is a widely used colorimetric method for determining the radical scavenging capacity of plants and extracts. This technique, which is based on the stable synthetic radical DPPH, is precise, simple to use, and inexpensive. This approach involves screening the overall activity of antioxidants. When DPPH combines with an antioxidant, it loses its ability to function as a free radical and turns yellow instead of violet [60].

It has been demonstrated that nSiO_2_ enhances plants' ability to withstand abiotic stress [61]. To reduce PEG-induced drought stress in *Mentha* plants, SiNPs were used as exogenous growth regulators in the present study. SiNPs at 50 ppm significantly enhanced the number of shoots, shoot height, FW, and DW. Similarly, Hongyan et al. [62] reported that following nSiO_2_ application, there was a considerable increase in root length; plant height; leaf area; and DW of the leaf, stem, and root because, at times of stress, the application of silica nanoparticles (NPs) accelerated plant development, and the NPs supplied more nutrients. Previous research has shown that wheat development under drought stress is enhanced by soil-amended SiNPs. Previous research by Esmaili et al. [63] has shown that the application of nanosilicon enhances the amount of active endogenous GA, which may help to explain why plants grown in higher silicon concentrations grow shorter and produce more lateral branches. Similar results were obtained in this study, as the SiNP concentration increased as the number of shoots increased with decreasing shoot height.

SiNPs may reduce the buildup of ROS in plants by stimulating the antioxidant defense system [64, 65]. SiNPs increase antioxidant activity and lower ROS levels in wheat [66].

Hongyan et al. [62] reported that nSiO_2_ can strengthen antioxidant mechanisms and potentially mitigate the consequences of oxidative stress. Under normal conditions, the antioxidant system of plants was not significantly affected by SiO_2_ treatment. Similar results were obtained in our study, as the RA content increased significantly in response to increasing SiNP application and then decreased at the highest SiNP concentration, and the total phenol content and scavenging activity were enhanced at 50 ppm SiNPs. The increase in plant tolerance to stress may be due to the strong antioxidase activity of nSiO_2_, and antioxidant activities can be controlled by the application of exogenous nSiO_2_, which also protects plants from oxidative damage [62]. According to several studies, silicon and silicon nanoparticles (SiNPs) have a beneficial impact on metabolic processes by acting as nanocarriers for proteins and nucleotides [67]. Due to Si-NPs' special characteristics, they can withstand abiotic stress and agricultural harm brought on by climate change. Si-NP reduced the impact of abiotic stress on the plant's fresh weight, chlorophyll content, photosynthetic rate, and leaf water content. It was found that 1 mM Si-NPs significantly increased the rate of photosynthetic transpiration under salinity stress of 50 mM [68]. Our results are in the same line with Rastogi et al. [68] and the findings of Avestan et al. [37], and Khattab et al. [38] who mentioned that adding SiO2-NPs to the MS medium enhanced the growth parameters and photosynthetic pigments of apple and *L. officinalis* respectively.

Manokari et al. [69] mentioned that SiNPs may be suspended in *in vitro* growth media of *Thunbergia erecta* (Benth.) T., where they enter the shoot system through cut ends and exhibit enhanced nutrient absorption to enhance morphometric growth characteristics. As the midrib parenchyma tissue density increases after receiving SiNP treatment, the cells are grouped compactly with well-developed vascular tissues, and the leaves exhibit enlarged vascular tissues with differentiated proto- and meta-xylem and phloem components. The addition of SiNPs to the growth media increases shoot proliferation and elongation and directly affects gene expression, which in turn affects plant defense mechanisms and stimulates shoot proliferation *in vitro*. SiNPs have been shown to promote structural changes in a variety of plant species. Thus, under field conditions, plants treated with SiNPs could tolerate drought stress [69]. Rastogi et al. [68] reported that nanosilicon may lessen transpiration in plants, increasing their heat, and drought resistance. Materials such as SiO_2_-NPs as a new alternative tool should be further studied for their potential to mitigate the negative effects of drought stress to serve as protective agents and raise the quantitative and qualitative characteristics of various crops in stressed or non-stressed situations by improving the efficiency of the components. Drought-tolerant plants can be selected using *in vitro* culture screening which can take a year or two in field experiments, the tolerant genotype was quickly and precisely identified 15 days following the use of the osmotic solution and SiO_2_-NPs treatment [35]. According to Khattab et al. [38] 50 mg/L of Si-NPs enhanced *L. officinalis* multiplication more effectively than 100 mg/L of Si-NPs. In the present study, compared with those of the control, the addition of 50 ppm SiNPs in combination with 10% PEG to the *M. pulegium* tissue culture medium promoted good growth, development, and antioxidant activity. However, as the SiNP concentration increased, both the quantitative and qualitative proliferation efficiency decreased.

GC–MS analysis of the *M. pulegium* methanol extract revealed the presence of 22 significant bioactive compounds, including fatty acids, antioxidants, alkaloids, and terpenes, with a variety of therapeutic, medicinal, and antibacterial qualities. Among the bioactive substances included in the methanol extract are monoterpenes such as limonene, linalool, pulegone, and camphor, and monoterpenic alcohols such as geraniol and menthol were shown to have antioxidant properties with anti-inflammatory and anticancer effects [9].

Khattab et al. [38] stated that Beta-linalool increased most in plantlets of *Lavandula angustifolia* cultivated in *in-vitro* conditions after adding 50 mg/L Si-NPs to the growth media. Our results are in agreement with their results as the principal constituents of the extract were menthol (59.73, 65.43%), geraniol (6.49–8.77%), linalool (3.84–4.64%), hexadecanoic acid methyl ester or methyl palmitate (3.2, 4.71%), pulegone (3.76, 2.76%), and limonene (2.51–2.99%) for the 0 ppm SiNPs, PEG 0% and 50 ppm SiNPs, and PEG 10%, respectively. Similar results were obtained by Beghidja et al. [3] working on *M. pulegium* in Algeria, except that the main component of the oil in Algeria was pulegone, which reached 43.3–87.36% in some cultivars; however, in the present study, the main component of the extract was menthol, reaching 59.73 and 65.43% for 0 ppm SiNPs, 0% and 50 ppm SiNPs, and 10% PEG, respectively, revealing that the chemical composition (chemotype) of *M. pulegium* varies according to its geographic distribution and cultivation environment. Similar results were obtained by Sharma et al. [70], who reported that the primary constituent of *Mentha arvensis L*. oil determined via GC–MS analysis was menthol (21.33%). According to Boga et al. [71], ajmaline is an antiarrhythmic medication used to treat acute ventricular or atrial tachycardia. Farnesol, found at 0.26 and 0.31% for 0 ppm SiNPs, 0% PEG, 50 ppm SiNPs, and 10% PEG, respectively, is a sesquiterpene alcohol with anti-inflammatory and anticancer properties. It can also modify different tumorigenic proteins by downregulating the expression of interleukin-6 in humans [72].

Most of the chemical compounds detected in the methanol extract using GC–MS were more abundant in the 50 ppm SiNP and 10% PEG treatment groups than in the control group. This difference might be due to the ability of elicitors to promote physiological and biochemical reactions that trigger the plant's defense mechanism and act as signals that are recognized by elicitor-specific receptors found on plant cell membranes and cause defensive reactions that boost the synthesis and storage of bioactive metabolites [21]. *In vitro* experiments can replicate the field environment, where plants are systematically subjected to unfavorable conditions, the study of abiotic stress through these studies is seen as entirely appropriate [35].

## Conclusion

The results presented in this study showed that the morphological and chemical characteristics were inversely proportional to the PEG concentration, as the highest PEG concentration (10%) had the lowest results. SiNPs (50 ppm) significantly enhanced all the morphological and chemical characteristics, while the parameters decreased at the highest SiNP concentration (100 ppm), except for the DPPH scavenging percentage, as there was no significant difference between the 50 and 100 ppm SiNPs. The best treatment for most of the parameters was 50 ppm SiNPs combined with 10% PEG. A phytochemical assessment of the *in vitro*-produced *Mentha pulegium* methanol extract was also conducted. These findings consistently demonstrated that *Mentha pulegium* methanol extract is a valuable source of physiologically active compounds with antibacterial and antiradical activity and highlighted the important role of PEG and SiNPs as elicitors in enhancing bioactive metabolites, suggesting its potential use in combination with pharmaceuticals to treat pathogenic bacteria either as a preventative measure or as a therapeutic agent.

### Supplementary Information

Supplementary Material 1.

Supplementary Material 2.

## Data Availability

This published paper and the supplementary data contain all the data created or analyzed during this investigation, also two additional files 1 and 2 are uploaded with the manuscript.
