# Supplementary material for: Enhancing growth and bioactive metabolites characteristics in Mentha pulegium L. via silicon nanoparticles during in vitro drought stress
Source: BMC Plant Biol. 2024 Jul 10;24:657. doi: 10.1186/s12870-024-05313-z (PMC11234791; doi:10.1186/s12870-024-05313-z)
Supplement: Supplementary file 1 — Supplementary Material 1. [file 12870_2024_5313_MOESM1_ESM.docx]

**Additional file 1:**

**Supplementary Tables S1: Absorbance of the standard (Gallic Acid).**

| Concentration of gallic acid (mg/ml) | Absorbance (nm) |
| --- | --- |
| 1 | 0.25 |
| 5 | 0.358 |
| 10 | 0.57 |
| 15 | 0.64 |
| 20 | 0.741 |
| 25 | 1.08 |

Absorbance of gallic acid as a standard

**Supplementary Tables S2.** The interaction effects of polyethylene glycol (PEG) and silicon nanoparticles (SiNPs) at different concentrations on the morphological and chemical characteristics of *M. pulegium*. LSD 0.05 = least significant difference at 0.05 probability.

| PEG  (%) | SiNPs  (ppm) | % of shoot formation | Days for shoot formation | Number of shoots | Shoot height  (cm) | Fresh weight (mg) | Dry weight (mg) | Chlo a  (mg/g FW) | Chlo b  (mg/g FW) | Rosmarinic acid (mg/gm FW) | Total phenolic (mg/g) | DPPH % |
| --- | --- | --- | --- | --- | --- | --- | --- | --- | --- | --- | --- | --- |
| 0%  5%  10% | **0** | 72.06 c | 25.00 b | 2.33 bc | 7.26 d | 1247.33 b | 120.00 bc | 0.76 d | 0.26 de | 0.010 d | 10.16 d | 33.46 e |
|  | **0** | 79.26 b | 27.66 a | 1.33 d | 6.46 e | 726.833 g | 73.866 e | 0.69 e | 0.19 e | 0.015 d | 13.14 c | 44.91 cd |
|  | **0** | 58.60 d | 28.33 a | 2.00 cd | 7.03 de | 1003.33 d | 126.66 b | 0.82 cd | 0.32 cd | 0.013 d | 13.29 c | 47.41 cd |
| 0%  5%  10% | **25** | 80.93 b | 18.66 e | 2.66 b | 7.43 cd | 1000.60 d | 116.66 bc | 0.83 cd | 0.34 cd | 0.012 d | 12.24 cd | 38.98 de |
|  | **25** | 79.43 b | 23.66 bc | 1.66 d | 6.76 e | 953.60 de | 97.066 d | 0.68 e | 0.18 e | 0.023 cd | 17.05 b | 49.03 c |
|  | **25** | 72.76 c | 23.33 c | 2.33 bc | 7.36 cd | 1133.5 c | 113.33 c | 0.86 bc | 0.36 c | 0.030 b | 16.29 b | 47.40 cd |
| 0%  5%  10% | **50** | 93.16 a | 18.66 e | 3.66 a | 9.03 a | 1383.83 a | 143.33 a | 0.92 b | 0.42 b | 0.033 ab | 16.583 b | 34.63 e |
|  | **50** | 82.00 b | 19.33 de | 2.66 b | 8.00 bc | 920.16 e | 110.10 c | 0.72 de | 0.22 e | 0.034 ab | 19.77 a | 42.59 d |
|  | **50** | 90.33 a | 20.33 d | 3.33 a | 8.36 b | 1427.16 a | 140.00 a | 1.24 a | 0.51 a | 0.039 a | 20.01 a | 82.37 a |
| 0%  5%  10% | **100** | 77.06 bc | 24.66 bc | 2.66 b | 7.93 bc | 826.96 f | 90.00 d | 0.81 cd | 0.31 d | 0.014 d | 9.93 d | 48.08 cd |
|  | **100** | 59.33 d | 23.00 c | 2.00 cd | 6.53 e | 906.66 e | 73.66 e | 0.59 f | 0.13 f | 0.016 d | 8.76 d | 74.99 b |
|  | **100** | 67.66 c | 28.00 a | 2.00 cd | 7.76 c | 833.76 f | 86.66 d | 0.78 d | 0.28 d | 0.022 cd | 11.51 cd | 31.80 e |
| LSD | | **6.51** | **1.55** | **0.55** | **0.43** | **68.07** | **10.78** | **0.065** | **0.048** | **0.0067** | **2.25** | **5.94** |

LSD_0.05_ = least significant differences at 0.05 probability. Means with the same letters in the same column are not significantly different (P ≤ 0.05) according to Tukey’s test.
