# Supplementary material for: Enhancing growth and bioactive metabolites characteristics in Mentha pulegium L. via silicon nanoparticles during in vitro drought stress
Source: BMC Plant Biol. 2024 Jul 10;24:657. doi: 10.1186/s12870-024-05313-z (PMC11234791; doi:10.1186/s12870-024-05313-z)
Supplement: Supplementary file 2 — Supplementary Material 2. [file 12870_2024_5313_MOESM2_ESM.docx]

**Additional file 2:**

**Supplementary Fig. S1** *Mentha pulegium* L.


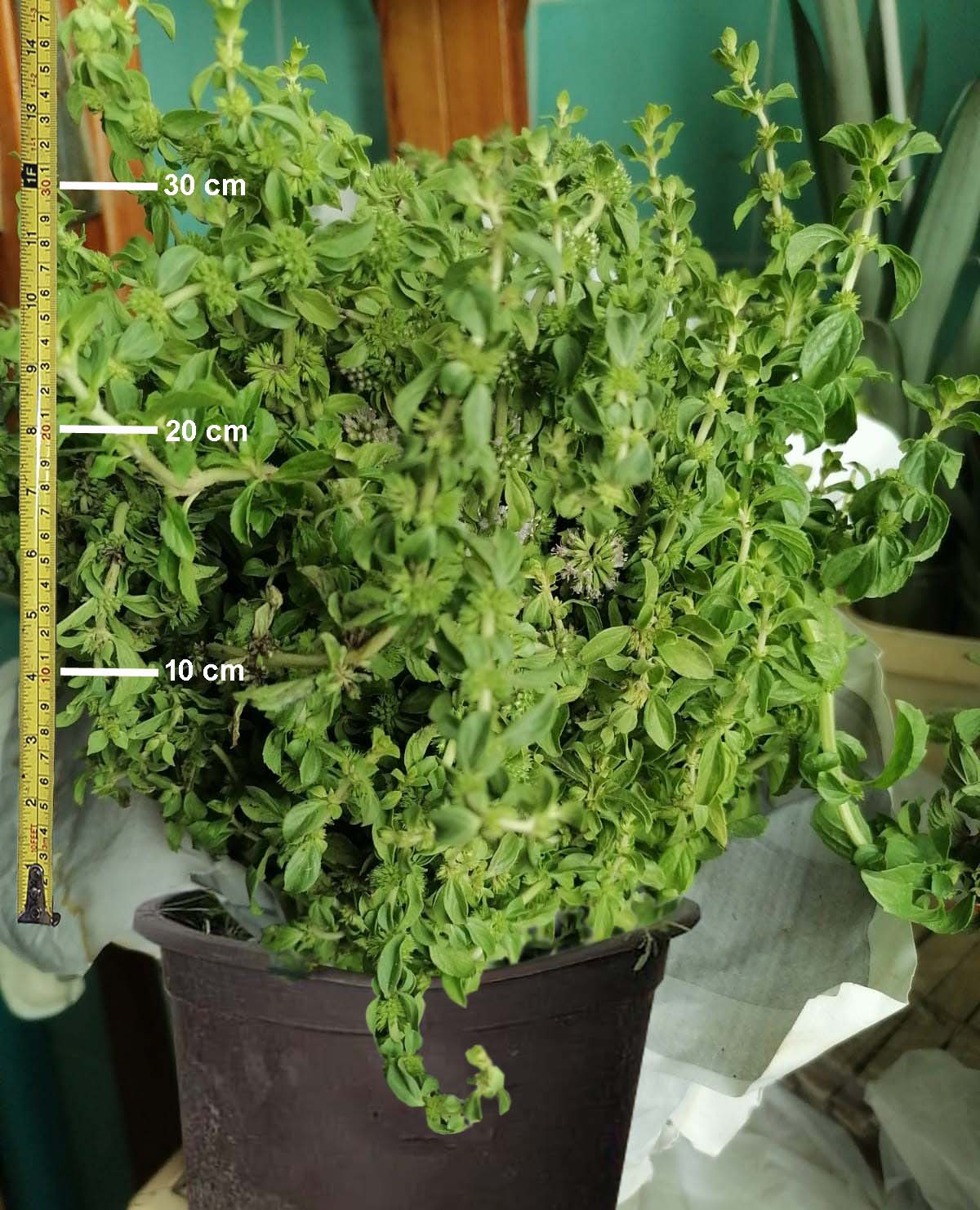


**Fig. S2** (**a)** Flowering branch of *Mentha pulegium* L.


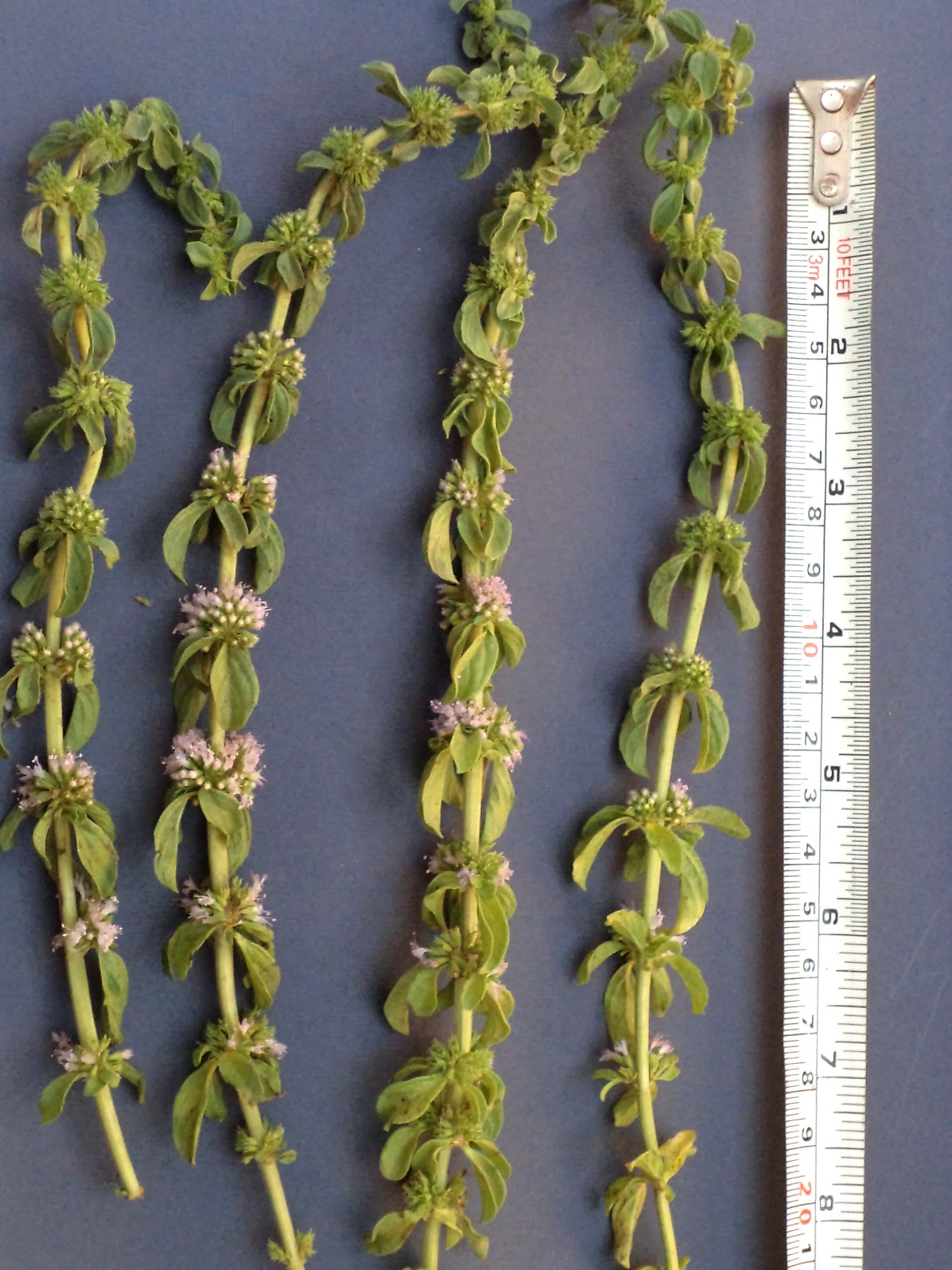


**Fig. S3**
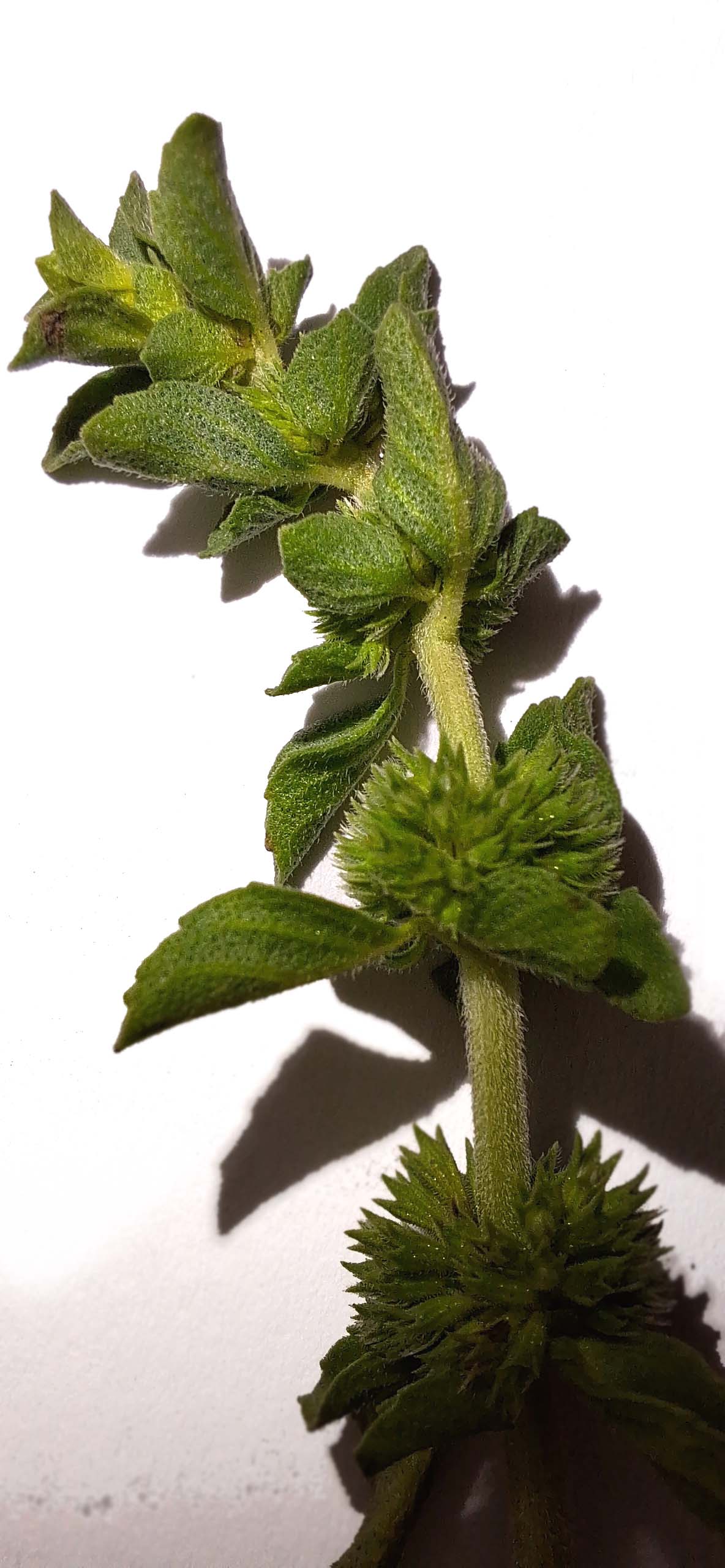
 Leafy branch and the inflorescences of *Mentha pulegium* L. before full opening.


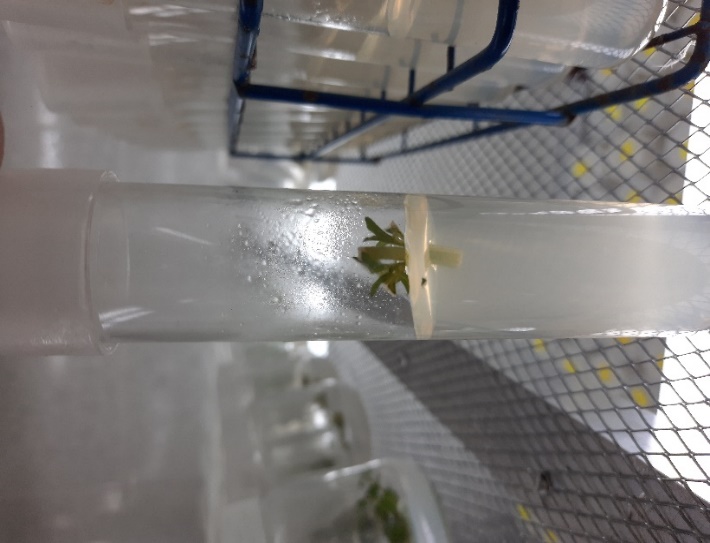

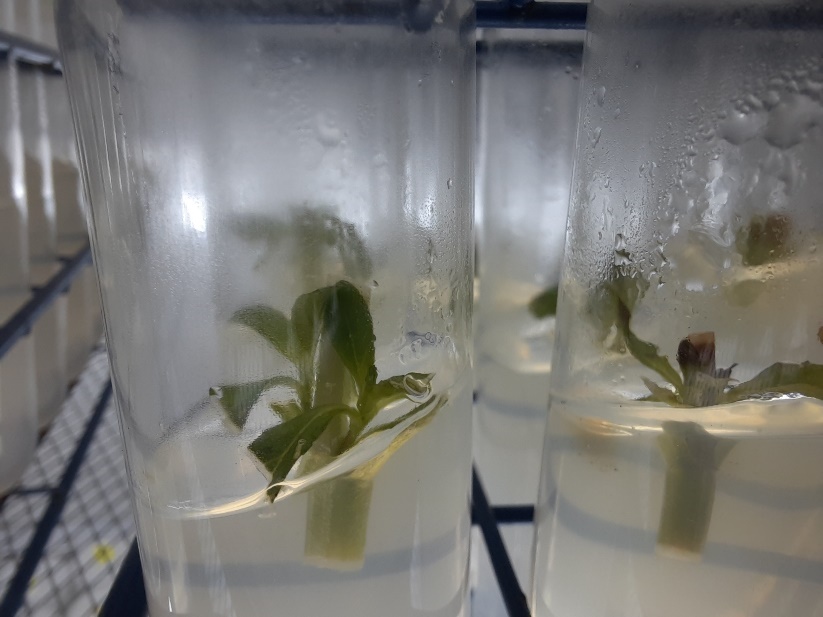
**Fig. S4** Fourth or fifth nodes from the growing point were removed and used as explants


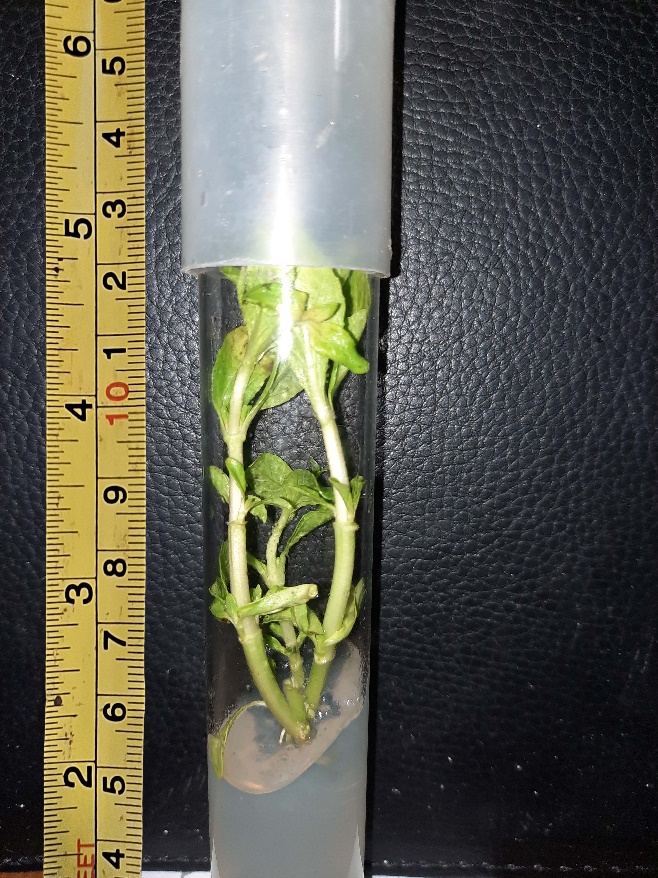
**Fig. S5** *Mentha pulegium* on MS medium supplemented with10% PEG, 50 ppm SiNPs, and the beginning of root formation
